# An asymptomatic *WASF1* truncation reveals pathogenic mechanism and therapeutic strategy for neurodevelopmental disorders

**DOI:** 10.3389/fnbeh.2026.1822055

**Published:** 2026-06-04

**Authors:** Shaojuan Song, Zongli Yang, Juanchun Gao, Xiang Wang, Yajing Zhang, Jiaxuan Wang, Shuying Yang, Jiayu Chen, Pu Wang

**Affiliations:** 1 Basic Medical College of Changzhi Medical College, Changzhi, China; 2 The First Clinical College of Changzhi Medical College, Changzhi, China

**Keywords:** asymptomatic truncation, lead compound, neurodevelopmental disorders (NDDs), virtual screening, *WASF1*

## Abstract

**Introduction:**

Wiskott–Aldrich syndrome protein family member 1 (*WASF1*) truncating variants, such as c.1516C>T (p.Arg506Ter), are established causes of neurodevelopmental disorders (NDDs), but their underlying pathogenic mechanism remains debated. This study aimed to clarify the disease mechanism and identify potential therapeutic leads.

**Methods:**

We characterized a novel, asymptomatic *WASF1* truncating variant (c.873delA) and compared its clinical and molecular consequences with those of the known pathogenic c.1516C>T variant. To target the likely pathogenic mutant protein, we performed high‑throughput virtual screening of the ZINC20 database.

**Results:**

Despite a similar reduction in wild‑type protein levels, the c.873delA variant did not cause neurological symptoms, in contrast to c.1516C>T. This observation supports a dominant‑negative, gain‑of‑function, or altered protein function mechanism rather than simple haploinsufficiency; however, the precise mechanism could not be definitively resolved from the available genetic and protein expression data. Virtual screening identified ZINC000101023849 as a high‑affinity lead compound with favorable drug‑like properties.

**Discussion:**

This study provides key evidence that *WASF1*‑related NDDs likely arise from a non‑haploinsufficiency mechanism and delivers a promising chemical lead for targeted therapy development. Further studies are needed to confirm the exact pathogenic mechanism and to validate the therapeutic potential of the identified compound.

## 1 Introduction

Wiskott–Aldrich syndrome protein family member 1 (*WASF1*) dysfunction is associated with diverse neurodevelopmental disorders (NDDs), exhibiting a broad phenotypic spectrum ranging from mild to severe intellectual disability (ID), autism spectrum disorder (ASD), epilepsy, and developmental delay (DD) (Kim et al., 2025). In 2018, Ito et al. (2018) reported that truncating variants in *WASF1* are linked to ID and epilepsy, with the c.1516C>T [p. Arg506Ter] variant specifically identified as the strongest causal factor in the affected individuals. A subsequent report in 2021 further corroborated this finding by documenting a recurrent *WASF1* truncating variant (c.1516C>T [p. Arg506Ter]) in a 6-year-old Japanese female patient, reinforcing the association between this rare yet recurrent variant and severe neurodevelopmental impairments (Shimojima Yamamoto et al., 2021).

*WASF1* is a regulatory factor abundantly expressed in the brain, particularly in neurons, oligodendrocyte precursor cells (OPCs), and mature oligodendrocytes (Kim et al., 2025). *WASF1* forms the WAVE regulatory complex (WRC) by assembling with four other protein families (such as Abl interactor, ABI; NCK-associated protein 1, Nap1; cytoplasmic FMR1-interacting protein, CYFIP; and hematopoietic stem cell protein 300, HSPC300). Under basal conditions, the WRC remains inactive. The activation of WRC occurs under two mechanisms:

1) Rac-mediated activation: Binding of the Rho-family GTPase Rac to Site A on the N-terminal region of CYFIP1 disrupts the autoinhibitory conformation of WRC, triggering structural rearrangement of *WASF1* and exposure of its WASP homology 2 (WH2) domain.
2) ADP-ribosylation factor 1 (ARF1)-coordinated activation: Rac binding to the Site D on the C-terminal region of CYFIP1 enhances CYFIP1’s affinity for the ARF GTPase ARF1. Their interaction indirectly induces WRC activation (Yang et al., 2022; Ding et al., 2022; Chen et al., 2017).

When WRC is activated, it regulates actin dynamics through interaction with the actin-related protein 2/3 (Arp2/3) complex. The Arp2/3 complex, an evolutionarily conserved actin nucleation center, undergoes conformational changes upon activation, enabling binding to the side of pre-existing actin filaments (mother filaments). Upon attachment, Arp2 and Arp3 nucleate the assembly of new actin branches (daughter filaments) (Goley and Welch, 2006; Rottner et al., 2021; Cao and Way, 2024). Actin plays critical roles in both neurodevelopment and mature neural function. In neurons, actin constitutes the primary cytoskeletal component, facilitating synaptic vesicle trafficking, anchoring vesicles at presynaptic terminals, driving dynamic morphological changes in dendritic spines, and maintaining postsynaptic protein organization and stability (Shimojima Yamamoto et al., 2021; Cingolani and Goda, 2008; Haseena et al., 2026). Dendritic spines, actin-rich protrusions extending from neuronal dendrites, serve as postsynaptic compartments for most excitatory synapses and mediate excitatory synaptic transmission in the brain (Han and Ko, 2023; Matus et al., 2000). Given the regulatory role of WRC and its interaction network in actin dynamics, coupled with the indispensable functions of actin in the nervous system, *WASF1* emerges as a pivotal player in neural homeostasis (Srivastava et al., 2021). Dysfunctional variants in *WASF1* may thus lead to a spectrum of neurological disorders.

Virtual docking technology, a cornerstone of computer-aided drug design (CADD), has demonstrated transformative potential in modern drug discovery. Advances in computational power and algorithmic optimization have significantly enhanced the accuracy and efficiency of virtual docking, enabling its critical role in novel drug discovery, target identification, and molecular optimization (Touati et al., 2024). In this study, molecular docking simulations were performed using AutoDock Vina, an open-source computational tool specifically designed to predict binding interactions between small-molecule ligands and biomacromolecules (such as proteins) (Trott and Olson, 2010; Morris et al., 2009).

The experiment is based on the hypothesis that the c.1516C>T [p. Arg506Ter] truncating variant may exert pathogenicity through a dominant-negative, gain-of-function, or altered protein function mechanism, as suggested by the genetic and protein expression data. We conducted large-scale virtual screening combined with computer-aided prediction to identify a potential lead compound from the ZINC20 database (http://zinc.docking.org/, a freely accessible commercial compound repository maintained by the Department of Pharmaceutical Chemistry at the University of California, San Francisco, containing three-dimensional (3D) structural data of 1.3 billion purchasable compounds). This compound exhibited high predicted binding affinity to the *WASF1* truncating variant (c.1516C>T [p. Arg506Ter]) and favorable physicochemical parameters, thereby facilitating subsequent rational drug design for therapeutic intervention.

## 2 Materials and methods

### 2.1 Asymptomatic truncated variant experiment analysis

#### 2.1.1 Clinical background

The proband was a 15-month-old girl referred to the Medical Genetics Center due to the presence of multiple café-au-lait spots (more than 1 cm in diameter) on the trunk and thighs. This presentation initially raised clinical suspicion for neurofibromatosis type 1 (NF1). Given the phenotypic heterogeneity of disorders presenting with café-au-lait spots and the need to evaluate multiple potential monogenic diagnoses simultaneously, whole-exome sequencing (WES) was conducted as a comprehensive first-tier diagnostic test. WES analysis did not identify a pathogenic variant in NF1. However, incidentally a novel heterozygous truncating variant in *WASF1* (NM_003931.3:c.873delA) was identified. Intriguingly, although this variant was classified as ‘likely pathogenic’ for NDDs based on the ACMG guidelines, neither the proband nor her variant-carrying father and grandmother exhibited any neurological symptoms typically associated with pathogenic *WASF1* variants. This discrepancy between the genetic finding and the asymptomatic presentation provided a unique opportunity to investigate the mechanism of *WASF1*-related pathogenicity.

#### 2.1.2 Sample collection and genomic DNA extraction

Following informed consent, peripheral blood samples were collected from the proband and her family members. Genomic DNA was extracted using a standard midi-volume blood genomic column kit and subsequently verified for quality (A260/A280 ratio ≥1.8) and integrity using a Qubit 2.0 Fluorometer and agarose gel electrophoresis.

#### 2.1.3 Whole-exome sequencing (WES) analysis

WES genomic DNA from the proband was subjected to sequencing to analyze the coding regions of approximately 20,000 functional genes in the human genome. Exome capture was performed using the xGen Exome Research Panel v2.0 (Integrated DNA Technologies, USA), targeting a total length of 42.5 Mb. High-throughput sequencing generated approximately 11,900 Mb (11.9 Gb) of raw data per sample. The sequencing data demonstrated high quality and coverage metrics: 99.70% of the target region was successfully covered, with 99.10% of the target bases achieving a sequencing depth of at least 20×. The base-calling accuracy was high, with Q20 and Q30 scores reaching 99.0% and 97.0%, respectively.

Bioinformatics analysis and variant interpretation raw sequencing data were processed and analyzed using a specialized cloud-based platform for the precise diagnosis of genetic diseases. This system integrates molecular annotation, biological analysis, and clinical interpretation. Variants were filtered and annotated against multiple authoritative databases, including pathogenic mutation databases, population frequency databases, Online Mendelian inheritance in man (OMIM), and MedGen. Variant classification and pathogenicity assessment were conducted in accordance with ACMG standards and guidelines (Richards et al., 2015), incorporating a three-element classification system (integrating genetic mode, phenotypic correlation, and variant function) to categorize genetic variations based on their clinical significance.

Sanger sequencing validation candidate pathogenic variants identified by WES were validated using the Sanger sequencing. Target regions containing the variants of interest were amplified using the polymerase chain reaction (PCR). The PCR products were purified and sequenced on an ABI 3730 DNA Analyzer (Applied Biosystems, USA). The resulting sequence chromatograms were analyzed using sequence analysis software to confirm the presence of the variants.

#### 2.1.4 Western blot analysis

A family-matched healthy control was included in this study, consisting of the proband’s paternal grandfather. The Sanger sequencing confirmed that the grandfather carries the wild-type WASF1 allele. Despite his advanced age, a sperm sample was successfully obtained, providing a genetically matched control to evaluate the specific impact of the mutation on WASF1 protein expression. Sperm was chosen as a proxy cell because *WASF1* is highly expressed in the testis and its role in cytoskeletal regulation is evolutionarily conserved, providing a sensitive system to detect protein truncation and stability. While sperm may not fully recapitulate the complex neuronal environment of the brain, this accessible tissue allowed the study to collect critical, family-matched protein-level data from the asymptomatic carrier and control that would otherwise be unattainable.

Total protein from both the proband’s father and the grandfather control was extracted using RIPA lysis buffer (Beyotime, Shanghai, China). The membranes were then incubated with primary antibodies, including an anti-WASF1 N-terminal antibody (Santa Cruz, sc-365165) and an anti-WASF1 C-terminal antibody (Santa Cruz, sc-271506), both diluted 1:1,000, and incubated overnight at 4 °C. Following primary antibody incubation, the membranes were washed again with tris-buffered saline with Tween-20 (TBST) six times, each for 5 min. Subsequently, the membranes were incubated with a secondary antibody conjugated with horseradish peroxidase (HRP) against rabbit or mouse IgG at a dilution of 1:5,000, and incubated at 4 °C for 12 h. Protein levels were assessed using the ChemiDOC™ XRS + imaging system (Bio-Rad). Olympus Image-Pro Plus software assisted in the quantitative analysis of the protein bands. Immunoblotting images were digitally captured using the same imaging system. Antibodies used in the protein blot analysis included anti-WASF1 N-terminal antibody (Santa Cruz, sc-365165), anti-WASF1 C-terminal antibody (Santa Cruz, sc-271506), and goat anti-mouse IgG heavy and light chains (HRP) (Abcam, ab205719).

### 2.2 Virtual screening based on the target protein

#### 2.2.1 Ligand database, docking software, and methods

Given that both the c.873delA and the pathogenic c.1516C>T variants are predicted to produce C-terminally truncated proteins that may exert pathogenicity through a similar dominant-interfering mechanism, we conducted a structure-based virtual screening targeting the c.1516C>T mutant protein to search for high-affinity lead compounds.

Furthermore, ZINC20 database was selected to retrieve small molecules as ligands and used OpenBabel for splitting (O’Boyle et al., 2011). Since AutoDock Vina can be executed through the command line or through Python makes it particularly suitable for batch processing of data and automation tasks. Apart from this, a Python script was used to enable the use of AutoDock Vina on a server for the high-throughput docking of proteins with small molecules, and to output the docking results sorted from highest to lowest score.

#### 2.2.2 Absorption, distribution, metabolism, excretion, and toxicity (ADMET) prediction

**Figure 1:**
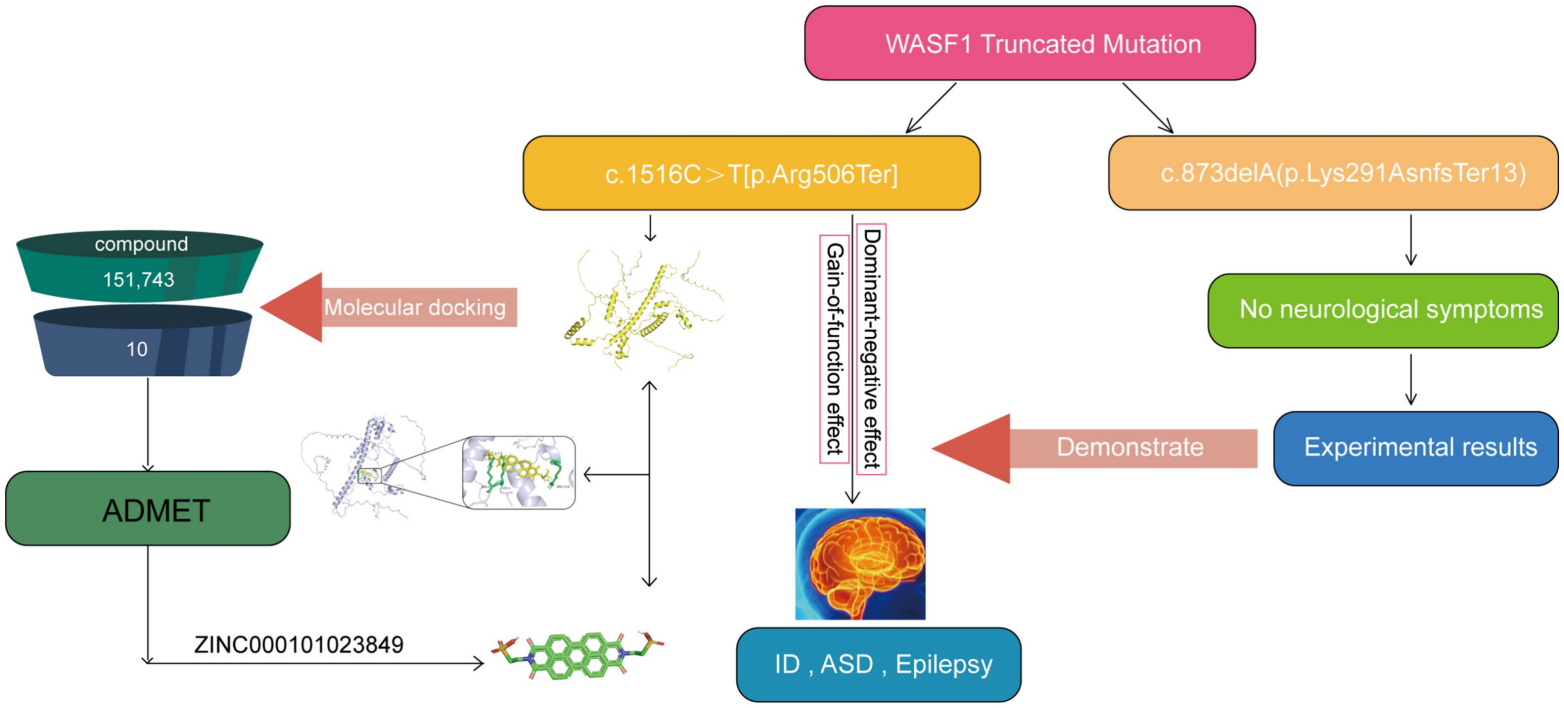
Flowchart depicting experimental process.

The ADMET properties of the selected compound were evaluated using SwissADME and ProTox-3.0 (Daina et al., 2017; Banerjee et al., 2024). This study also analyzed the water solubility, metabolism, blood–brain barrier (BBB) permeability, cytochrome P4502D6 (CYP2D6), hepatotoxicity, neurotoxicity, and mutagenicity of the selected compound (see Figure 1).

## 3 Results

### 3.1 Clinical information

**Figure 2:**
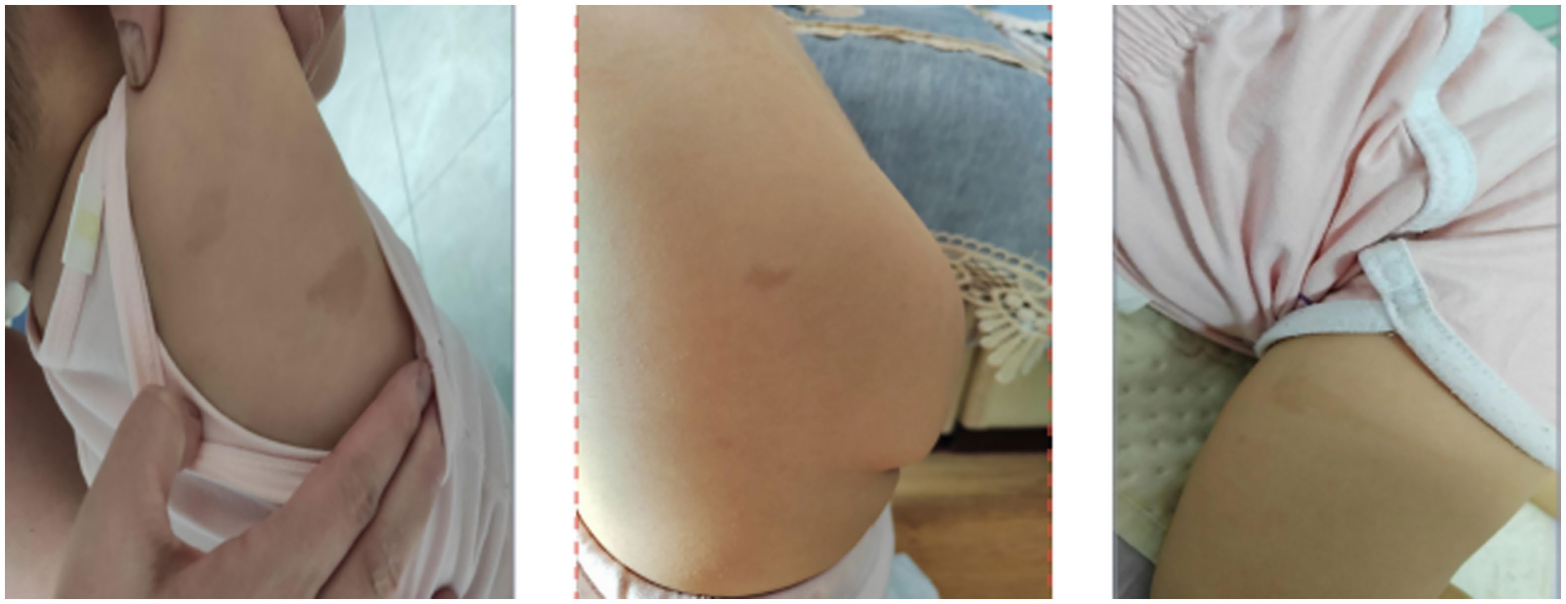
Café-au-lait spots on the trunk and thighs.

**Figure 3:**
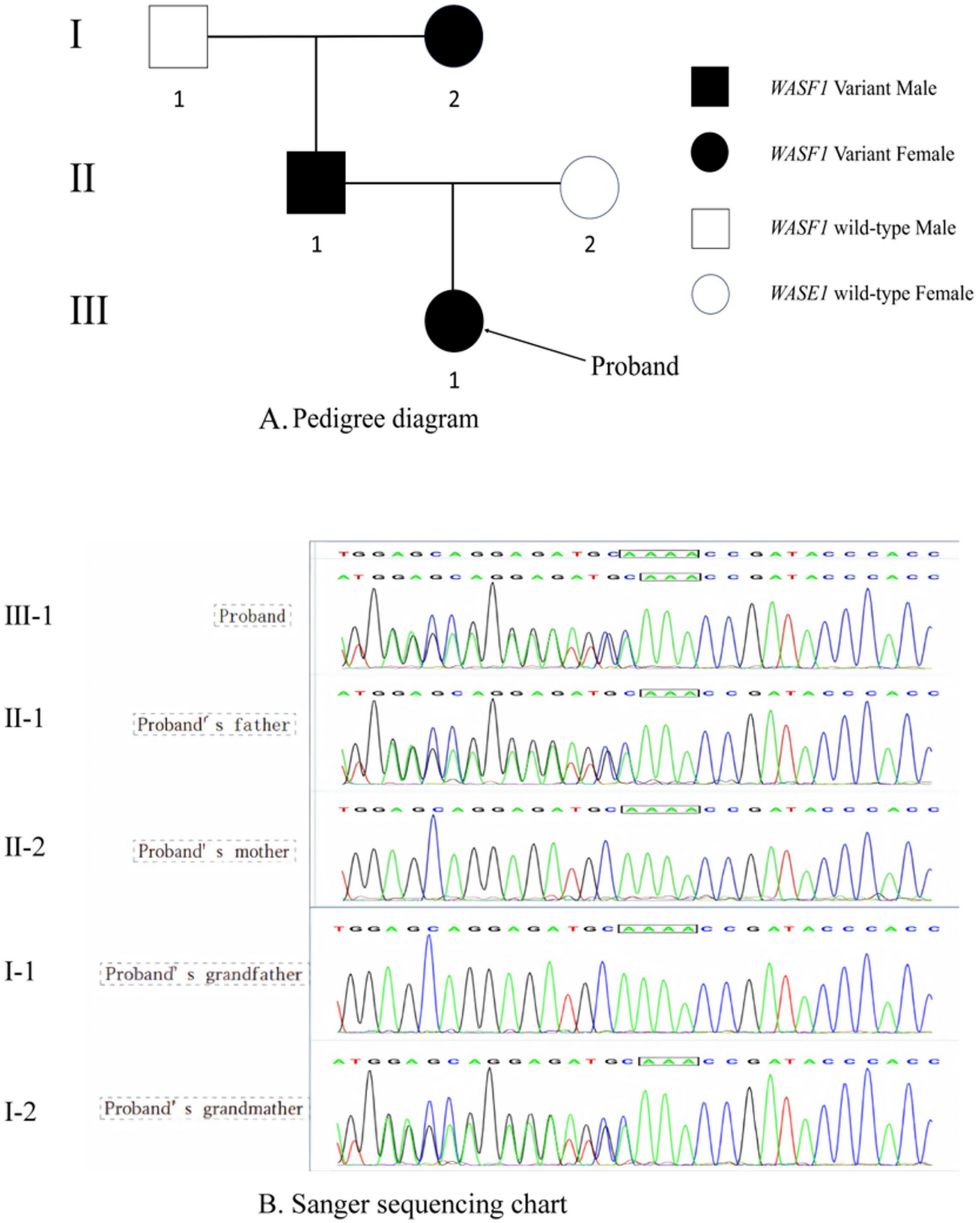
**(A)** Pedigree showing incomplete penetrance of the *WASF1* c.873delA variant, and **(B)** Sanger sequencing chart of *WASF1* variant sites in the proband’s family (variant sites are indicated in the corresponding boxes).

The clinical information included the following:

(i) Initial symptoms and onset: The proband presented with multiple café-au-lait spots.
(ii) Reason for current presentation: The patient was referred for evaluation of these cutaneous findings.
(iii) Prior diagnostic and treatment history: Before this referral, no specific diagnostic evaluation (such as dermatological assessment, neuroimaging, or prior genetic testing) or treatment had been performed for the café-au-lait spots.
(iv) Other relevant history (such as birth, developmental, and family): The patient was born at full term through an uncomplicated delivery with normal birth parameters. Her developmental milestones were age-appropriate: gross motor (sat without support at 7 months, walked independently at 13 months), fine motor (pincer grasp established by 10 months), language (3–5 meaningful words by 14 months), and social skills (responds to name). There was no history of seizures, hypotonia, or developmental regression. Family history was significant: the proband’s father and paternal grandmother, later confirmed to carry the same *WASF1* variant, were clinically asymptomatic with no neurological symptoms or café-au-lait spots (Figure 3A).
(v) Detailed physical examination findings: Physical examination revealed multiple café-au-lait spots (>1 cm) on the trunk and thighs (Figure 2). A detailed neurological examination was normal, with no dysmorphic features, ocular abnormalities, hypotonia, bradykinesia, or other focal deficits noted.
(vi) Auxiliary Examinations: Routine blood tests, including complete blood count and basic metabolic panel, were within normal limits. Given the absence of neurological symptoms or signs on examination, specific investigations such as brain magnetic resonance imaging (MRI), computed tomography (CT), or electroencephalography (EEG) were not clinically indicated at the time of presentation.
(vii) Indications for Genetic Testing: The presentation of multiple café-au-lait spots initially raised suspicion for neurofibromatosis type 1 (NF1). To broadly investigate monogenic causes, WES was performed as a comprehensive first-tier diagnostic test.
(viii) Genetic Testing Results: WES did not identify a pathogenic variant in NF1. However, the test revealed a novel heterozygous truncating variant in the *WASF1* (NM_003931.3(*WASF1*):c.873delA[p. Lys291AsnfsTer13]). Copy number variation sequencing (CNV-seq) showed no significant deletions or duplications, and the Sanger sequencing confirmed this variant in the proband and identified the same in her asymptomatic father and paternal grandmother, establishing a hereditary pattern (Figure 3B).
(ix) At 15 months of age, the proband as of now presents without neurological symptoms. However, given the variable age of onset reported in WASF1-related NDDs, continued longitudinal follow-up may be essential to determine whether this represents a truly asymptomatic carrier state or a delayed phenotypic manifestation.

### 3.2 Genetic and functional analysis of the *WASF1* c.873delA variant

**Figure 4:**
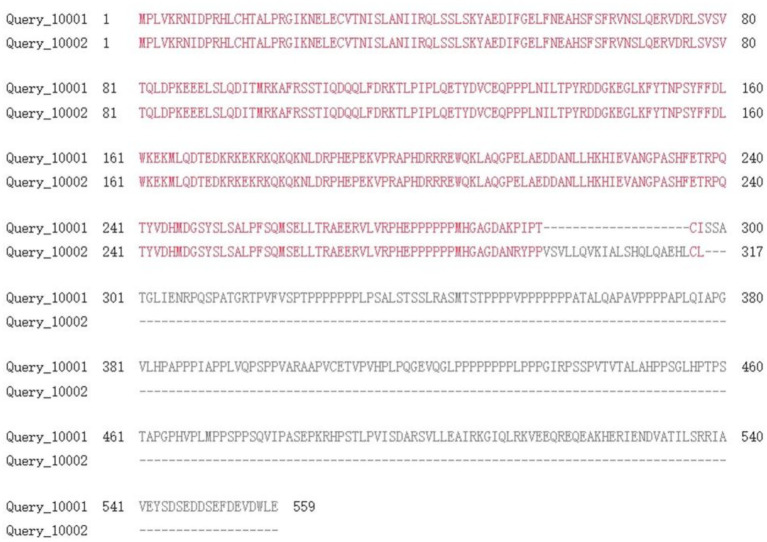
NCBI prediction comparison chart of wild-type and variant protein sequences (red indicates the same sequence).

According to the guidelines of ACMG or the Association for Molecular Pathology (AMP), the c.873delA variant was classified as likely pathogenic (LP): pathogenic very strong-criterion 1 (PVS1) + pathogenic moderate-criterion 2 (PM2). The PVS1 criterion was applied because the frameshift variant introduces a premature termination codon (p.Lys291AsnfsTer13), predicting a truncated protein and loss-of-function (LOF) in a gene where LOF is a well-established disease mechanism. The PM2 criterion was met as the variant is absent from major population frequency databases (such as gnomAD and dbSNP). The wild-type WASF1 protein is 559 amino acids long, with a critical WH2 domain located between residues 494–521 (Figure 4). The c.873delA variant is predicted to result in a premature stop codon at position 13 of the new reading frame, truncating the protein upstream of this functional domain.

**Figure 5:**
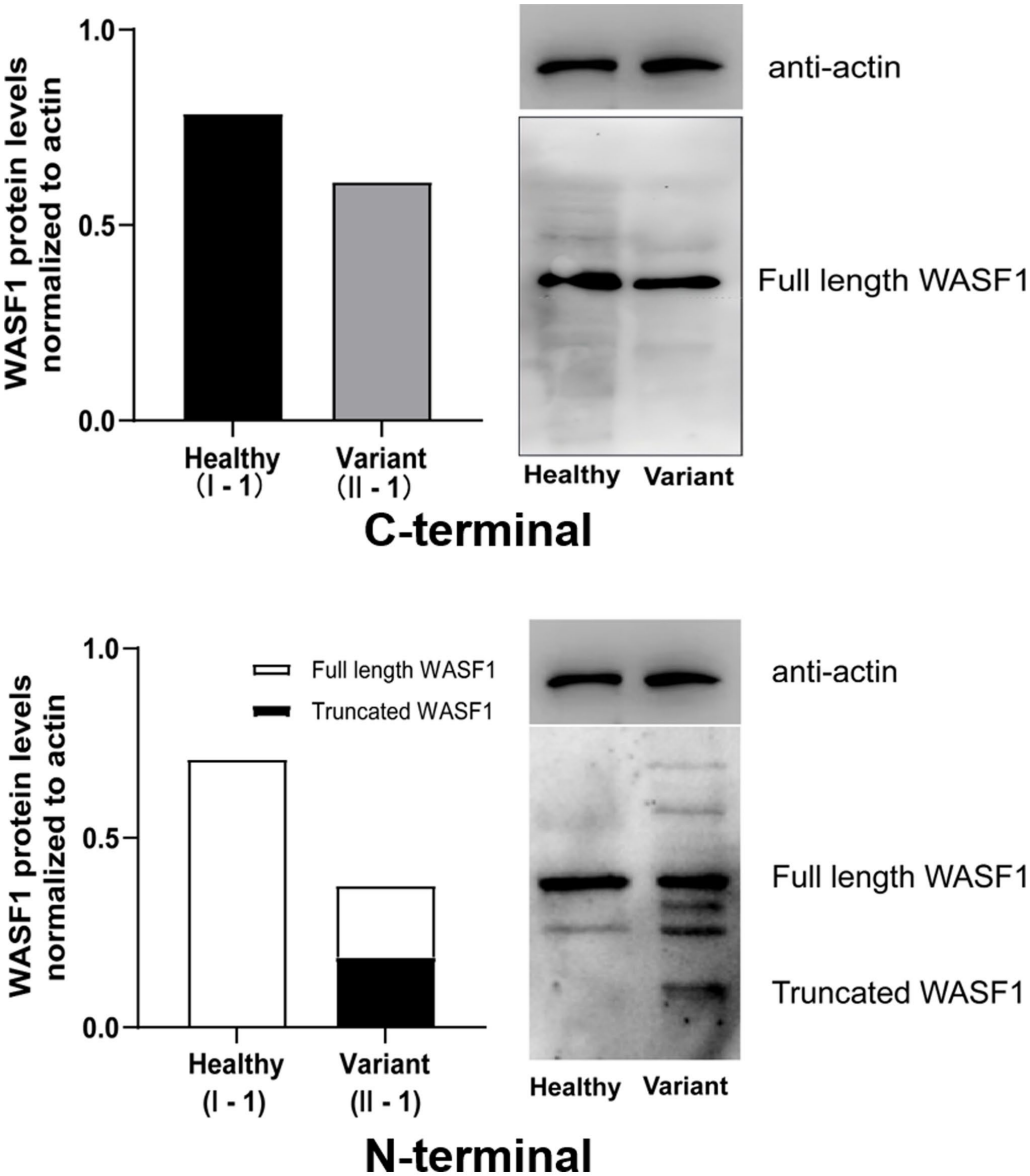
WB and densitometry quantification results.

To assess the variant’s effect on protein expression, we performed the Western blot analysis on sperm samples collected from the proband’s father (a heterozygous carrier) and grandfather, who served as a family-matched wild-type control. Despite the grandfather’s advanced age, a sperm sample was successfully obtained. Sanger sequencing confirmed the wild-type genotype in the grandfather and the heterozygous status in the father (Figure 3B). Using an antibody targeting the N-terminal region of *WASF1*, we detected both the full-length *WASF1* protein (~75 kDa) and a shorter truncated form (~35 kDa) in the carrier, a finding absent in the control samples (Figure 5). Densitometry quantification indicated that the full-length protein level in the carrier was reduced to approximately 50% of the wild-type control, while the truncated isoform was present at about 20% of the control’s full-length levels. This confirms that the variant leads to the production of a stable, truncated protein isoform rather than complete nonsense-mediated decay of the transcript.

### 3.3 Virtual screening of potential compounds

#### 3.3.1 Docking score results

First, we retrieved 151,743 small molecules from the ZINC20 database and performed molecular docking within the docking pocket of the target protein. Each docking output file contains multiple poses with corresponding scores. Generally, the pose with the highest score in each file was considered optimal. The top 10 compounds listed according to higher docking scores, are presented in Table 1.

**Table 1:** Top 10 compounds ranked using AutoDock Vina.

Number	Compound	AutoDock Vina Score (kcal/mol)
1	ZINC000072266819	−9.4
2	ZINC000253395968	−9.1
3	ZINC000101023849	−9.0
4	ZINC000408977682	−8.9
5	ZINC000001680522	−8.9
6	ZINC000059639459	−8.9
7	ZINC000016940894	−8.9
8	ZINC000012374509	−8.8
9	ZINC000032109805	−8.8
10	ZINC000100962632	−8.7

Strong binding: ≤ − 9 kcal/mol; Medium binding: –7 ~ –9 kcal/mol; Weak binding: ≥ − 5 kcal/mol.

#### 3.3.2 Absorption, distribution, metabolism, excretion, and toxicity (ADMET) prediction

**Figure 6:**
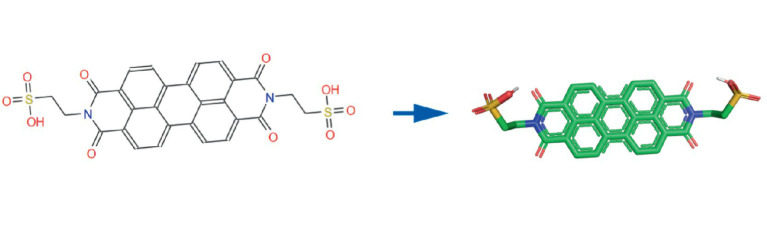
Chemical structure of ZINC000101023849.

**Figure 7:**
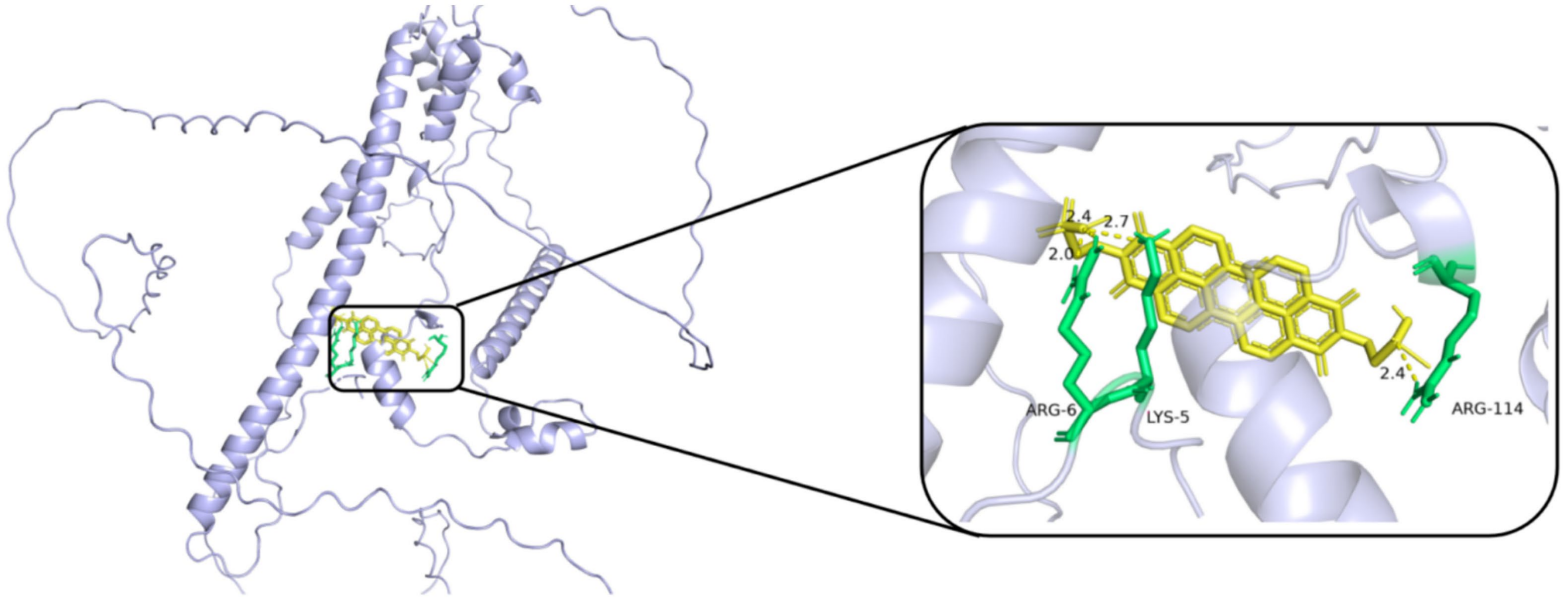
Visualization of docking results.

We utilized SwissADME and ProTox-3.0 to predict the ADMET properties of the selected small molecules. The results indicated that ZINC0000101023849 has low metabolism, does not inhibit CYP2D6, possesses blood–brain barrier (BBB) permeability, and show no evidence of hepatotoxicity, neurotoxicity, or mutagenicity, making it an ideal compound. In contrast, the other nine molecules have moderate to high levels of neurotoxicity, hepatotoxicity, and mutagenicity, effectively eliminating them from further drug development and testing (see Figures 6, 7; Table 2).

**Table 2:** Absorption, distribution, metabolism, excretion, and toxicity (ADMET) properties of drugs.

Number	Compound	Metabolism^a^	CYP2D6^b^	BBB^c^	Hepatotoxicity^d^	Neurotoxicity^e^	Mutagenicity^f^
1	ZINC000072266819	H	F	F:0.97	0	2	0
2	ZINC000253395968	H	F	T:0.63	0	2	1
3	ZINC000101023849	L	F	T:0.65	0	0	0
4	ZINC000408977682	H	F	T:0.84	0	2	0
5	ZINC000001680522	H	T	T:0.78	0	1	1
6	ZINC000059639459	H	F	T:0.78	0	1	0
7	ZINC000016940894	H	F	T:0.76	1	2	2
8	ZINC000012374509	L	F	T:0.74	0	1	2
9	ZINC000003219805	H	F	T:0.74	1	1	0
10	ZINC000100962632	L	F	T:0.68	0	1	0

a Metabolism: L (Low) or H (High).

b CYP2D6: T (Inhibitory) or F (Non-inhibitory).

c Blood–brain barrier (BBB) Penetrating: T (True) or F (False).

d Hepatotoxicity: 0 (None), 1 (Low-moderate), or 2 (Moderate-high).

e Neurotoxicity: 0 (None), 1 (Low-moderate), or 2 (Moderate-high).

f Mutagenicity: 0 (None), 1 (moderate), or 2 (high).

## 4 Discussion

The identification of WASF1 truncating variants as a cause of NDDs has established a clear genotype–phenotype association (Ito et al., 2018). However, the underlying pathogenic mechanism—whether haploinsufficiency or a dominant-negative/gain-of-function effect of the truncated protein—remains debated (Dahl et al., 2003; Kessler, 2025). The discovery of the asymptomatic c.873delA variant in this study provides a critical genetic experiment that challenges and refines this mechanistic understanding.

The data present a fundamental paradox: a novel, earlier-truncating variant (c.873delA) that is predicted to produce a protein of 290 amino acid residues followed by 12 mutated residues—significantly shorter than the established pathogenic variant (c.1516C>T, p.Arg506Ter)—yet results in no discernible neurological phenotype across three generations. Notably, the asymptomatic carriers in this pedigree include two adults (the father and grandmother), effectively ruling out delayed onset or age as confounding factors. The Western blot analysis confirmed that carriers express approximately 50% of wild-type *WASF1* alongside a stable truncated isoform. This finding provides direct evidence that the presence of a normally structured and functional *WASF1* allele on the homologous chromosome is sufficient to prevent NDDs and epilepsy, even when the other allele produces a severely truncated isoform. This decisively argues against haploinsufficiency as a sufficient disease mechanism, as a 50% reduction of wild-type protein is compatible with normal neurological function.

The resolution to this paradox lies in the precise site of truncation. The wild-type WASF1 protein contains a critical C-terminal WCA domain, which comprises the Verprolin (V/WH2), Cofilin (C), and acidic (A) regions (Padrick et al., 2011). Within this domain, the WH2 motif (residues 494–521) is particularly essential for Arp2/3 complex activation (Alekhina et al., 2017; Sridharan Iyer et al., 2025), as the activation is responsible for binding monomeric actin and promoting the formation of branched actin filaments (Nakamura et al., 2011). Furthermore, the activation maintains the dynamic structure of dendritic spines, thereby supporting synaptic signal transmission and plasticity (Haseena et al., 2026; Soderling et al., 2003). The c.1516C>T variant truncates the protein precisely within this WH2 motif (at residue 506), potentially generating a stable fragment that retains the structural determinants necessary to interact with the WRC or Arp2/3. We hypothesize that the specific fragment may act as a “poison” subunit, capable of sequestering or misdirecting components of the WRC or the Arp2/3 complex. In stark contrast, the c.873delA variant truncates the protein upstream of all known functional domains. The resulting protein, although stable, appears to lack the structural determinants required for effective interaction with the WRC or Arp2/3, rendering it functionally inert. The benign nature of the c.873delA truncation suggests that the pathogenicity previously attributed to the c.1516C>T variant may not originate entirely from the truncation event. The NDDs and epilepsy observed in the reported Japanese cohort may therefore arise from a combination of variant-specific toxic properties and additional genetic or environmental modifiers.

This site-of-truncation hypothesis has direct implications for therapeutic strategy. When the phenotype in c.1516C>T patients is indeed driven, at least in part, by the unique toxic property of the truncated protein, then therapeutic efforts should focus on directly targeting and eliminating that mutant protein, (e.g., through degradation technologies like PROTACs). The structure-based virtual screening, which identified ZINC000101023849 as a high-affinity lead compound for the c.1516C>T mutant protein, represents a rational first step towards such a variant-specific therapeutic approach. Conversely, strategies aimed solely at augmenting wild-type WASF1 expression may be less effective when the primary pathology is driven by the mutant protein’s interfering activity.

We acknowledge two primary limitations that outline essential directions for future research. First, direct functional assays are needed to compare the biochemical and cellular impacts of the c.873delA and c.1516C>T truncated proteins on WRC assembly, actin polymerization, and neuronal morphology, to empirically test the structural hypothesis. Second, the therapeutic potential of ZINC000101023849 requires validation in both *in vitro* and *in vivo* models of the c.1516C>T mutation. Despite these necessary steps, the genetic evidence from the asymptomatic c.873delA carrier family provides a pivotal insight: it necessitates a paradigm shift from evaluating truncation variants solely based on predicted loss-of-function, to a nuanced consideration of the structural and functional consequences of the specific truncation site. This framework may be crucial for accurate pathogenicity assessment and the development of mutation-specific therapeutic strategies for WASF1-related disorders.

## 5 Conclusion

This study leverages a unique asymptomatic *WASF1* truncation variant to provide genetic evidence that challenges the haploinsufficiency model for c.1516C>T-related *WASF1* NDDs. While the precise mechanism (such as dominant-negative, gain-of-function, or altered protein function) requires further functional validation, these findings suggest that therapeutic strategies directly inhibiting or degrading the mutant protein (especially through PROTAC technology) may be more appropriate than those aimed at augmenting wild-type protein expression. This mechanistic insight pivots the therapeutic strategy from protein supplementation to mutant protein inhibition and degradation. Using a structure-based virtual screening pipeline that integrates binding affinity and ADMET properties, this study has identified ZINC000101023849 as a high-affinity lead compound targeting the *WASF1* mutant protein. Furthermore, this study not only provides genetic evidence challenging the haploinsufficiency model but also pivots the therapeutic approach from protein supplementation to mutant protein inhibition, delivering a high-affinity lead compound for targeted therapy development.

## Data Availability

The raw data supporting the conclusions of this article will be made available by the authors, without undue reservation.
